# A Systematic Literature Review on Waste-to-Resource Potential of Palm Oil Clinker for Sustainable Engineering and Environmental Applications

**DOI:** 10.3390/ma14164456

**Published:** 2021-08-09

**Authors:** Ahmad Hussaini Jagaba, Shamsul Rahman Mohamed Kutty, Gasim Hayder, Lavania Baloo, Azmatullah Noor, Nura Shehu Aliyu Yaro, Anwar Ameen Hezam Saeed, Ibrahim Mohammed Lawal, Abdullahi Haruna Birniwa, Abdullahi Kilaco Usman

**Affiliations:** 1 Department of Civil and Environmental Engineering, Universiti Teknologi PETRONAS, Bandar Seri Iskandar 32610, Perak Darul Ridzuan, Malaysia; shamsulrahman@utp.edu.my (S.R.M.K.); lavania.baloo@utp.edu.my (L.B.); azmatullah_16005709@utp.edu.my (A.N.); nura_19001733@utp.edu.my (N.S.A.Y.); 2 Department of Civil Engineering, Abubakar Tafawa Balewa University, Bauchi 740272, Nigeria; Ibrahim.lawal@strath.ac.uk; 3 Department of Civil Engineering, College of Engineering, Universiti Tenaga Nasional (UNITEN), Kajang 43000, Selangor Darul Ehsan, Malaysia; 4 Department of Civil Engineering, Ahmadu Bello University, Zaria 810107, Nigeria; 5 Department of Chemical Engineering, Universiti Teknologi PETRONAS, Bandar Seri Iskandar 32610, Perak Darul Ridzuan, Malaysia; anwar_17006829@utp.edu.my; 6 Department of Civil and Environmental Engineering, University of Strathclyde, Glasgow G1 1XJ, UK; 7 Department of Chemistry, Sule Lamido University, Kafin-Hausa PMB 048, Nigeria; birniwa01@gmail.com; 8 Civil Engineering Department, University of Hafr Al-Batin, Hafr Al-Batin 31991, Saudi Arabia; aukilaco@uhb.edu.sa

**Keywords:** palm oil clinker, palm oil mill, engineering application, environmental management, waste utilization, systematic literature review

## Abstract

Several agro-waste materials have been utilized for sustainable engineering and environmental application over the past decades, showing different degrees of effectiveness. However, information concerning the wider use of palm oil clinker (POC) and its performance is still lacking. Therefore, as a solid waste byproduct produced in one of the oil palm processing stages, generating a huge quantity of waste mostly dumped into the landfill, the waste-to-resource potential of POC should be thoroughly discussed in a review. Thus, this paper provides a systematic review of the current research articles on the several advances made from 2005 to 2021 regarding palm oil clinker physical properties and performances, with a particular emphasis on their commitments to cost savings during environmental and engineering applications. The review begins by identifying the potential of POC application in conventional and geopolymer structural elements such as beams, slabs, and columns made of concrete, mortar, or paste for coarse aggregates, sand, and cement replacement. Aspects such as performance of POC in wastewater treatment processes, fine aggregate and cement replacement in asphaltic and bituminous mixtures during highway construction, a bio-filler in coatings for steel manufacturing processes, and a catalyst during energy generation are also discussed. This review further describes the effectiveness of POC in soil stabilization and the effect of POC pretreatment for performance enhancement. The present review can inspire researchers to find research gaps that will aid the sustainable use of agroindustry wastes. The fundamental knowledge contained in this review can also serve as a wake-up call for researchers that will motivate them to explore the high potential of utilizing POC for greater environmental benefits associated with less cost when compared with conventional materials.

## 1. Introduction

Agricultural production has grown more than threefold in the last 50 years as a result of soil expansion for agricultural use, the green revolution’s technological development, and stimulated population growth [1]. Agriculture has grown to be one of the biggest and most influential economic sectors in several countries, as well as the highest job creator on the planet. It generates an average of 23.7 million food tons per day around the world. This increase in global production has put more strain on the environment, resulting in negative repercussions on air, soil, and water resources, with later effects on public health and ecosystem sustainability. It is culpable for 21% of all greenhouse gas emissions. This prioritizes the development of a sustainable agriculture, with a focus on minimizing environmental effects through the use of innovative technological systems [2].

Agriculture is one of the biggest biological sectors, generating a lot of biomass, which becomes a major ingredient for the bioeconomy. This presents a huge opportunity as it promotes the conversion of vegetable waste into value-added products such as feed, bioproducts, food, and bioenergy. It also facilitates the lowering of fossil fuel utilization and greenhouse gas emissions. Agricultural waste is widely available, renewable, and inexpensive. Its application has the prospects to be environmentally friendly [3]. Its main structural components have high content of starch, hemicelluloses, cellulose, and lignin. Previously, these agricultural wastes were treated solely to adhere to environmental policy and regulations. In recent times, the lack of agricultural waste and the need for a more sustainable society have pushed for waste transformation into beneficial materials rather than simple treatment.

Agricultural vegetal wastes, also known as biomass, have a significant ability to produce sustainable energy from renewable fuels. They can be thermally and chemically treated to produce a variety of useful materials, including biofuels, biooils, biogases, and biosolids. Biomass can also be thought of as an energy storage medium. Cereal starch, primarily derived from wheat and maize, is primarily used as a raw material in the production of ethanol. To enhance the ignition characteristics of biomass wastes and convert them to liquid biofuels or combustible gaseous products, thermochemical treatments such as gasification, torrefaction, and pyrolysis are used. The largest global vegetable oils are derived from sunflower seed, cotton seed, olive, rapeseed, palm, peanut, soybean, and coconut. The most common wastewaters generated by the edible oil companies are palm oil mill effluent (POME) and olive oil wastewater (OMW) [4].

Olive oil mills represent one of the traditional agricultural crop production industries containing valuable compounds found in herbal extracts. Olive oil is the primary source of fat in the Mediterranean diet where its nutritional benefits are globally recognized for possible diabetes or cardiovascular disease prevention upon adequate consumption. It has a long history as traditional remedies in different cultures due to its health benefits, such as antioxidant [5], anti-inflammatory, antiaging, and antimicrobial properties, which are related to chronic disorders. Today, olive trees (*Olea europaea L*.), native to the Mediterranean basin, have spread to many countries solely because of the olive oil industry. This growth is associated with the health advantages of olive oil consumption, as this product is high in nutritional and bioactive compounds [6]. Olive oil production is important to the Mediterranean region’s economy. Its extraction produces a solid waste known as olive mill solid waste (OMSW), as well as a liquid part mixed with wash-waters known as OMWW. OMWW is slightly acidic, with high levels of polyphenols, suspended solids, and organics. Polyphenols are phytotoxic despite having health-promoting effects stemming from strong antioxidant properties. It has been proven by the literature that the negative effect of OMWW on the environment can attain the same levels as the total pollution produced by 22 million people per year [7]. The generation of olive mill waste seems to be significantly lower than that of the oil palm industry. Because of its high oil rate of return, low-cost palm oil is mostly used as a food ingredient or as a feedstock for the cosmetics, biodiesel, pharmaceutical, and oleochemical industries. In 2018/2019, worldwide vegetable oil production was approximately 203.7 Mt, with palm oil accounting for a significant portion (35.7%) [8].

**Figure 1:**
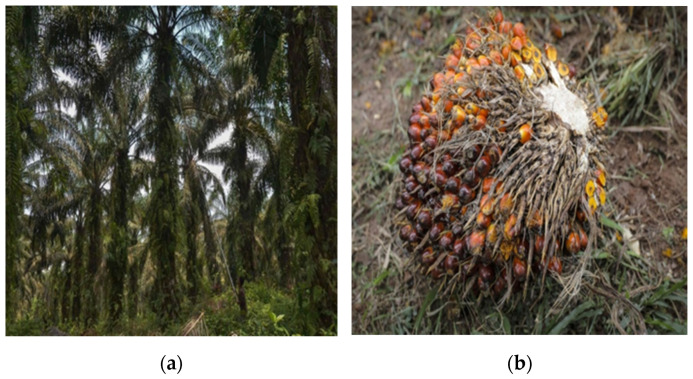
(**a**) An oil palm tree and (**b**) a palm oil seed bunch oil.

Palm oil has become a significant commodity in the world. The palm oil business is rapidly expanding and establishing itself as an important agriculture-based sector (see Table 1). It is one of the main agricultural crops that thrives in a hot tropical climate. It produces vegetable oil fit for human consumption. As depicted in Figure 1a,b, the tree is naturally brown and the seed is reddish in color due to a high beta-carotene content. Oil palm has demonstrated great value as almost every part of its plant is useful.

The palm oil industry significantly contributes to a producing country’s growth domestic product (GDP). Palm oil is a tasty vegetable oil that is extracted from the mesocarp of fruit from the oil palm species *Elaeis guineensis*. It is naturally reddish in color. A typical palm oil mill yields crude palm oil/kernels and biomass as primary and secondary products, respectively [9]. During wet milling, palm oil extraction discharges huge quantity of biomass wastes, consisting of oil palm trunks, empty fruit bunches, oil palm fronds, palm kernel shells, palm oil mill sludge, palm oil fuel ash, palm mesocarp/press fibers, palm sludge cake, palm oil mill effluent, and palm oil clinker (POC) [8]. The palm oil industry faces significant challenges in meeting the increasingly stringent environmental regulations on waste disposal. Therefore, the versatility of palm oil industry waste such as POC to be turned into different products and its potential as an energy source is expected to soon lead to greater demand. Moreover, the sustainability and environmental consequences associated with said waste material need to be addressed.

### Sustainable Approach for Oil Palm Waste Handling

In a global partnership, the United Nations member countries set 17 Sustainable Development Goals (SDGs) to enhance health and education, eliminate poverty, and stimulate economic growth through the use of renewable resources. Biomass is used in attempts to decrease the use of oil-based products to produce usable energy, value-added products, or energy vectors. Admittedly, biomass accounted for 12% of total energy consumed in 2018. As a result, numerous biomass sources, including lignocellulosic residues, energy crops, and woody biomass, have been explored as potential feedstocks to be upgraded via various conversion pathways [10]. However, biomass materials from certain crops causes harm to the environment, as well as decrease oil palm production yield.

In recent times, *Ganoderma* diseases, which are triggered by the white rot fungus *Ganoderma boninense*, have been a disturbing factor. They are the most serious threat to long-term oil palm production. Therefore, long-term bioremediation technologies that ameliorate soil ecosystems should be regarded as a disease control alternative [11]. The excessive use of chemical fertilizers has caused land degradation, with degraded soil conditions on most farms, and it has ultimately reduced yield and oil extraction rate. This has greatly affected the sustainability of the oil palm production by making replanting estates uneconomical and unsustainable. The greatest of all is the high CO_2_ emissions [12]. To avert this, the process necessitates a shift in mindset on the part of the economy’s producers and consumers. This very shift has the capability to persuade them to adopt eco-friendly practices. Just after legislation enacted by the European Union in 2018 to terminate feedstock utilization in biofuels that contribute to climate change, the European Commission published suggested criteria for determining which crops cause any harm. The use of more detrimental biofuels will be maxed at 2019 levels until 2023, then lowered to zero by 2030, according to the new EU law. This is to help in cutting carbon dioxide emissions by 45% and protecting the environment. However, it also states that producers who can demonstrate increased yields may be exempt. It can then be contended that their crops meet the demand for biofuel, food, and feed without the need for expansion into nonagricultural lands [13].

Despite the fact that EU has revised its biofuels policy to phase out palm oil-based biodiesel, original palm plantation nations would not suffer from this situation as the waste (empty fruit bunches, potash ash, palm kernel, fiber, shell, POME, decanter sludge, and POC) generated from the plantations can be widely used in the following aspects:

- Generation of biobased chemicals: furfural, 2,3-butanediol, lactic acid, succinic acid, levulinic acid, xylitol, and oxalic acid;
- Biochar production;
- Fertilizer and animal feed;
- Fermentation medium;
- Microalgal growth medium;
- Renewable source of carotene;
- Biopolymer production;
- Manufacturing of engineered wood products such as particle, composite, and fiber board;
- Reinforcing agent;
- Filler;
- Adhesive;
- Isolation of cellulose nanofiber, microcrystalline cellulose, and nanocrystalline cellulose;
- Pulp and paper production;
- Precursor material in coir fiber, roofing tile, card paper, and cement board manufacturing.

In general, the aforementioned aspects cover a wide range of industries such as human and animal food, pharmaceutical, cosmetics, fertilizer, construction, chemical and biotechnological processes, and water treatment plants. Therefore, it can be concluded that, even with the new EU policy, palm plantation nations would still survive.

**Table 1:** Palm oil production globally from 2016 to 2020 (USDA, 2020) [14].

	Thousand Metric Tonne					
Production	2016/2017	2017/2018	2018/2019	2019/2020	2020/2021 (July)	2020/2021 (Aug)
**Indonesia**	36,000	39,500	41,500	42,500	43,500	43,500
**Malaysia**	18,858	19,683	20,800	19,000	19,300	19,700
**Thailand**	2500	2780	3000	2800	3100	3100
**Colombia**	1146	1627	1632	1529	1670	1670
**Nigeria**	990	1025	1015	1015	1015	1015
**Other**	5845	5960	6077	5927	6013	6013
**Total**	65,339	70,575	74,024	72,771	74,598	74,998

**Figure 2:**
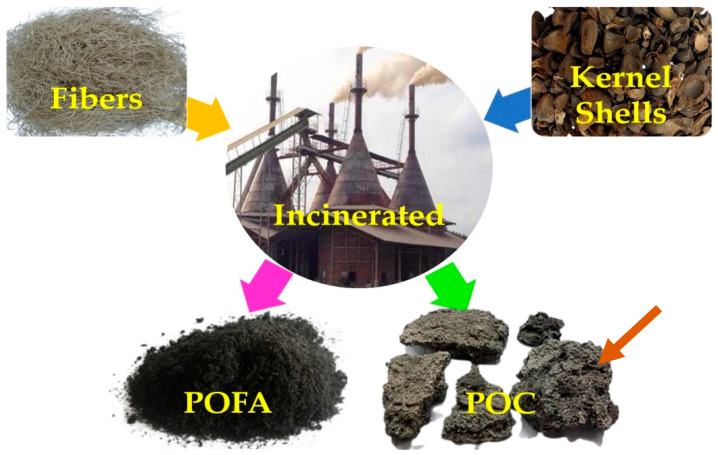
By-products generated from the burning of palm oil waste materials [19].

Palm oil clinker (POC) is a waste byproduct generated in one of the oil palm processing phases [15]. It is a residue from the heating zone of a steam boiler during electricity generation produced in huge amounts from the calcination of oil palm fibers and shells in a suitable proportion of 30:70 at 100–850 °C (see Figure 2) [16,17]. It is subsequently cooled at air atmosphere [18].

**Figure 3:**
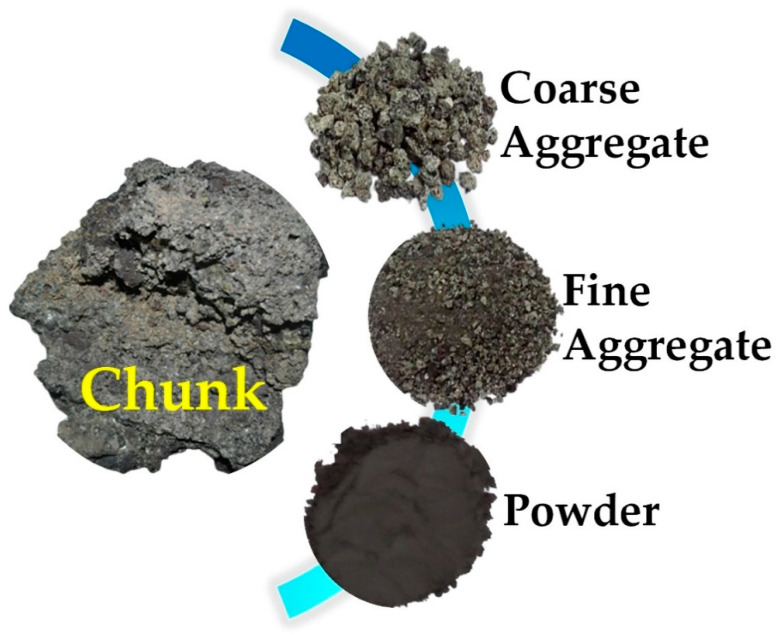
POC chunk, coarse aggregate, fine aggregate, and powder [28].

Physically, POC, as shown in different forms in Figure 3a–c, is naturally porous, flaky, gray in color, irregular in shape, and lighter in weight, with rough and sharp broken surfaces [20,21]. It is mostly presented as a solid lightweight material with a size between 2 and 15 cm [22,23]. POC is made up of inorganic oxides, 3.35% organic carbon, and minerals such as halite, lysite, eglestonite, elatossite, quartz, and cristobalite [24]. The chemical oxide composition of POC, as highlighted in Table 2, varies according to several factors. Some of these are fiber-to-shell ratio, applied incineration process temperature, palm tree location soil condition, and POC form (nano, powder, fine, or coarse particle size) [22,25]. POC is a pozzolanic aggregate capable of producing appropriate attachment in a geopolymer matrix because of the presence of alumina–silica compounds [26]. It is not surprising that pore structure has a close association with the resistance of cement-based substances to fluid infiltration. This property includes pore size distribution, interconnectivity, and porosity [27].

The oil palm extraction rate has rapidly increased due to the increasing oil palm global demand [39,40]. As a consequence, fresh porous lumped POC is continuously generated [41]. The produced POC in the boiler is mixed in suspension, moved from the combustion boiler, and deposited in the factory yard [34]. It is a solid waste product of little to no economic benefit that causes pollution of the atmosphere, soil degradation, and ground water contamination [24]. In recent times, it has mostly been dumped in landfills, which not only causes soil pollution but also goes as far as contaminating ground water [42]. Continuous disposal would result in waste accumulating at the dumpsite, necessitating the allocation of new space for landfills. This would have negative consequences for the environment as fertile land is converted into a refuse collection area [43]. 

In addition to overcoming waste disposal problems, integrating low-cost and environmentally friendly waste materials in new and sustainable product development would help in environmental pollution control, appropriate land use, and promoting sustainability [35,44]. Therefore, reusing POC for different applications would assist in preserving natural resources and reducing greenhouse gas emission, thereby paving the way for proper consumption and producing a cleaner environment [27,45]. With the growth of technology, there is a need to abandon the use of archaic outdated materials in industrial applications. Raw materials utilized by companies have a greater impact on the environment. It is critical to alter the current situation, particularly for emerging countries [29]. Many attempts have been made to develop low-carbon products or procedures in order to achieve the concept of green technology. POC emerges as a future viable industrial material with uses in several industries because of its outstanding mechanical qualities and environmental advantages. The physical, chemical, and microstructural properties of POC byproducts of palm oil are discussed in this article. Its goal is to provide a comprehensive overview of POC’s current and potential energy applications. A critical evaluation of its application in many fields is presented, as well as its modification via various physical and chemical processes. A new potential direction beyond the state of the art is the application of POC in nano form; accordingly, only one such article was found in the literature review.

## 2. Methodology

**Figure 4:**
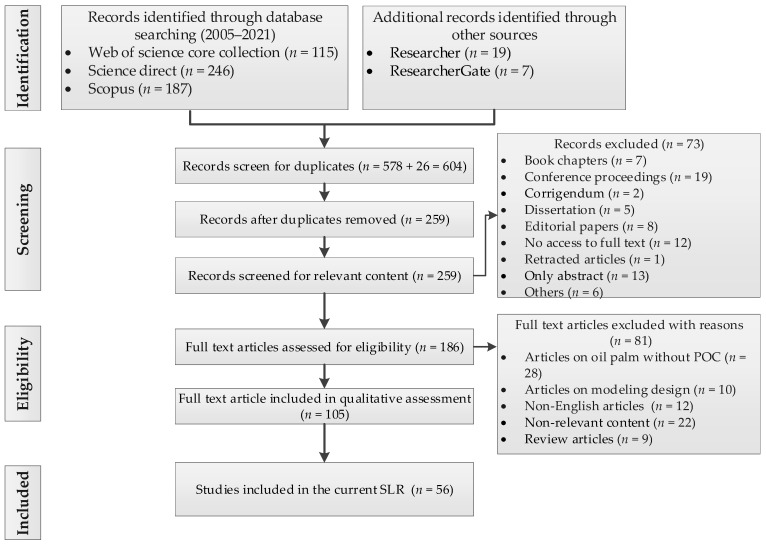
Flowchart for the PRISMA review process.

A systematic literature review (SLR) is a method of study that is specific and complete, carried out in a steady and particularized manner to support article choice and the screening of appropriate review articles [46]. It is a type of secondary investigation that identifies, selects, analyzes, and interprets all available information pertaining to a single area of study, research question, or research paradigm. The SLR described here started in early April 2021. The pattern used in this study for the SLR is depicted in Figure 4 (PRISMA). This would only be achievable if research questions related to the research objectives were identified.

### 2.1. Specific Research Questions

Through the large utilization of oil palm tree for oil palm production amounting to waste generation and subsequent environmental pollution, it has become necessary to study the reutilization of different waste product generated from the production process in palm oil mills. As a result, the primary goal of this research was to form an opinion of POC reusability potential for engineering and environmental applications. The study further highlights how POC pretreatment could enhance its reusability using different materials and become more significant than the normal POC without pretreatment.

The design of the review process in a typical SLR is frequently guided by research questions. As a result, the most significant aspect of any SLR is defining the questions and determining the best way to answer them. The primary goal of this SLR was to answer questions about the efficiency of POC during different applications in the engineering aspect. As a result, the research objectives and corresponding questions specifically formed for this study are shown in Table 3.

### 2.2. Criteria and Bibliographical Search Strategy

The search strategy used in this overview concentrated on four electronic resources to find journal articles relevant to the paper scope.

The databases were chosen because they are among the most comprehensive and exhaustive scientific databases with broad data coverage, thereby motivating the development of meaningful bibliometric enquiries. Other sources such as “Researcher” and “ResearchGate” were used to obtain extra records.

In terms of search standards, this review comprised the period 2005 to April 2021, including subsequent studies, given that several POC applications are still being investigated for use in engineering to improve waste reuse and save money. The data collection ended on 5 April 2021.

The very next module of the literature search was to insert two keywords in the document search to acquire POC investigations from the scientific community: “Oil Palm” AND “Palm Oil Clinker” were used as the TITLE-ABS-KEY. The term “POC” was omitted due to the fact that most authors do not use it in their article titles. Instead, they refer to it as “palm oil clinker”, because POC is an abbreviation. Lastly, the authors of this article agreed and used the “Tittle” search to find specific articles that focused on POC reusability, which is pretty much unavoidable in oil palm mills. Surprisingly, we could not find a single SLR article that looked into the waste-to-resource potential of POC for various engineering and environmental applications.

### 2.3. Screening

In this study, the screening stage began with the removal of duplicates. Due to the fact that four electronic databases were considered, as well as two additional sources, numerous matches were found. After excluding duplicates, the leftover records were screened for relevant content. Retracted articles, book chapters, corrigenda, no access to full text, dissertations, conference proceedings, editorial papers, abstract only papers, and others were excluded at this juncture.

### 2.4. Criteria for Article Exclusion and Inclusion

The ultimate search was carried out in order to obtain the most essential articles for use in the SLR detailed in this review. The study was carried out by targeting articles with valid research, clear contributions, and enriched data.

The exclusion and inclusion criteria were designed to find the optimal studies, as well as extract and synthesize the necessary data. Initially, a huge number of articles were gathered for analysis from the chosen databases. According to Equation (1), the prejudice for the article inclusion or exclusion baseline was determined as a function of the included articles’ relevance ratio (*R*) for a given year (*y*), the total number of proposed keywords (*n*), the number of initial papers in a given year (*P*), and the number of matched keywords (*k*).

(1) $$R^{y}=\frac{\sum_{i}^{n}\frac{k_{i}}{n}}{P^{y}}.$$

Equation (2) was utilized to support the inclusion and exclusion of articles in this study [47].

(2) $$f\left(k_{i}\right)=\{\{\begin{array}{cc} included, & R^{y}<\frac{k_{i}}{P^{y}}\\ excluded, & \left(otherwise\right) \end{array}\}.$$

The inclusion requirements for articles chosen for the full review after filtering due to their high quality and well-known importance are outlined in Figure 2.

### 2.5. Data Analysis

**Figure 5:**
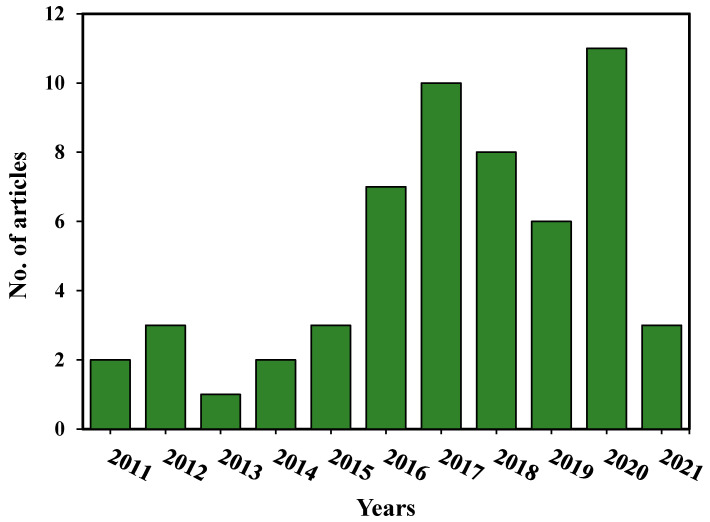
Published articles on POC per year.

Amidst advances in technology, interest in POC reutilization for engineering and environmental applications consistently increased from 2005 to 2021. However, one thing that may be of interest is that a variety of applications were investigated during the studied time frame. This was achieved because these materials were subjected to characterization, highlighting their properties before and after use. According to Figure 5, the year 2020 had the most articles with 11, followed by 2017 and 2018 with 10 and eight articles, respectively. It can also be seen that the year 2013 had the fewest articles with a single article.

**Figure 6:**
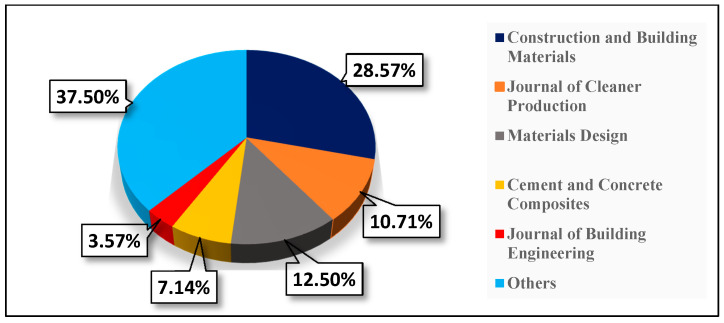
Percentage of articles published per journal.

Twenty-one different sources were evaluated in this SLR. This proves that POC is applicable in different aspects of engineering due to its potential impact on performance and cost saving. Surprisingly, only three sources published >5 articles, for a total of 29 articles. Of the three sources, the journal with 16 papers was “Construction and Building Materials”. The journal specializes in publishing research articles in the field of engineering materials for sustainable construction. The second most common journals were the “Journal of Cleaner Production” and “Materials Design”, having six and seven articles, respectively. Figure 6 shows that the journals “Cement and Concrete Composites” and “Journal of Building Engineering” had four and two articles, respectively.

Seven journals covering 21.5% of the total 56 articles examined in this SLR had one article each. These journals were as follows: Materials proceedings, Sains Malaysiana, Journal of Ecological Engineering, Resources, Conservation and Recycling, Jurnal Teknologi, European Journal of Environmental Civil Engineering, Water, Air, and Soil Pollution. Eleven journals each had one scientific paper on POC that met the inclusion criteria for this SLR. These were as follows: Materials (MDPI), Advanced Materials Research, International Journal of Integrated Engineering, IOP Conference Series: Materials Science and Engineering, Sustainability (MDPI), KSCE Journal of Civil Engineering, MATEC Web of Conferences, AIP Conference Proceedings, International Journal of GEOMATE, Indoor Built Environment, and Coatings (MDPI).

It is worth mentioning that, when evaluating these sources, most had a very sectoral reach with respect to engineering utilization of POC. Therefore, journals with a substantial waste, environmental, manufacturing, and construction background, such as the “Journal of cleaner production”, “Construction and building materials”, “Materials design”, and “Journal of building engineering”, are carving a widening niche in POC studies. 

Content analysis was utilized to identify the geographical locus and sectoral focus of the articles. Although neither term was included in the keywords, content analysis was conducted to categorize the articles in terms of location. It is interesting to observe that different countries were involved in studying POC materials, with13 countries across the globe identified.

**Figure 7:**
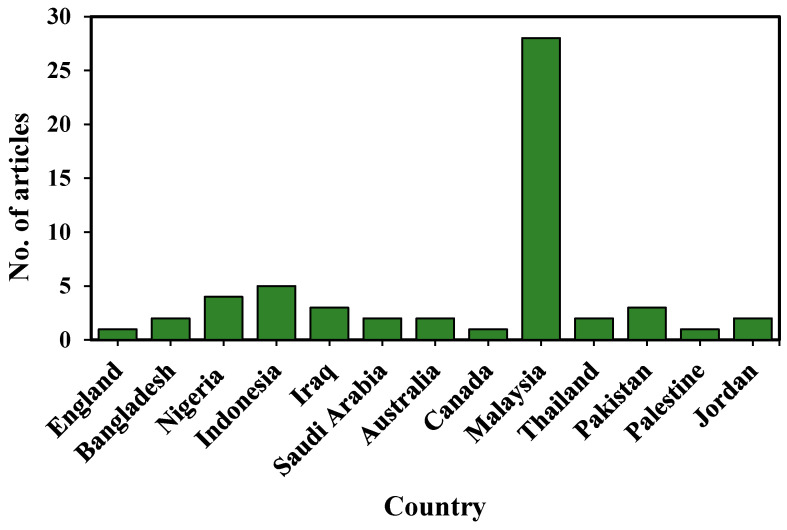
POC research studies across countries.

Malaysia, Indonesia, and Nigeria, with 28, five, and four articles, respectively, were the three countries with the highest number of published articles considered in this study (see Figure 7). Pakistan and Iraq published three articles each. Bangladesh, Saudi Arabia, Australia, Thailand, and Jordan published two articles each. Some countries published only one article each, such as England, Canada, and Palestine.

**Figure 8:**
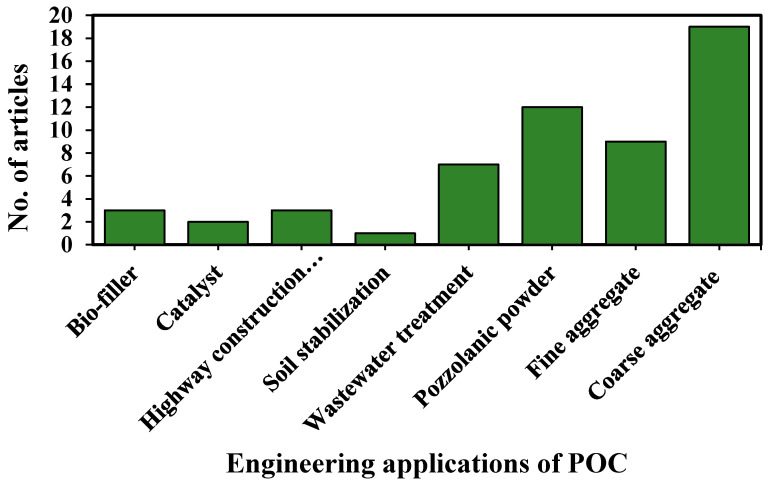
Articles published with various POC applications.

Figure 8 describes number of articles considered in this SLR according to engineering and environmental aspects of utilization. Bio-filler and highway construction materials had three articles each, catalysts had two articles, wastewater treatment had seven articles, pozzolanic powder material had 12 articles, fine aggregate had nine articles, coarse aggregate had 19 articles, and soil stabilization had only one article.

## 3. Waste-to-Resource Potential of POC

### 3.1. POC in Geopolymer Structural Elements

Geopolymer is an inorganic material that can be formed through the use of a binder. According to [48], any material that contains silica and alumina can be utilized as a binder. Alkali activators are also important for the production of geopolymers. Numerous high-silica and high-alumina waste materials can be utilized for geopolymer production due to their pleasing size, shape, and chemical composition. POC, considered a pozzolanic aggregate, has the capacity to create a good bond in a geopolymer matrix as it possesses the aforementioned characteristics (see Table 4). When comparing POC with OPC concrete, the use of a geopolymer binder increases the workability and strength of POC concrete, thus lowering its water absorbability. A green and long-lasting structural lightweight concrete can be produced by combining POC with a fly ash-based geopolymer binder [49]. Utilizing POC particles in the geopolymeric specimen results in structural elements with good resistance to water absorption.

Sustainability in high-strength concrete production can be achieved by combining POC with fly ash as a geopolymer-based binder. Designing and mixing concrete with 100% POC aggregate can give rise to a concrete with compressive strength >30 MPa and a density of 1821 kg/m^3^. However, a 32% strength reduction was experienced as natural aggregate was substituted by OPC. Furthermore, 75% POC aggregate replacement in geopolymer concrete mixtures was proven by [49] to be the most effective. As POC concentration increases in geopolymer concrete mixtures, water absorption increases as density decreases.

POC sand was used for full sand replacement in a geopolymer mortar and achieved comparable mechanical properties, showing high resistance to MgSO_4_ and HCl solutions. In this case, 53 MPa was recorded as the 28 day compressive strength with 17% density reduction [28].

A geopolymer concrete containing 100% POC as coarse aggregate was designed and evaluated. According to the results, 41.5 MPa was the highest compressive strength achieved at 28 days curing with a density range of 1910–2172 kg/m^3^. Splitting tensile strength increased and UPV values were also good. POC also improved the compressive toughness of the geopolymer mortar. The study concluded that structural-grade lightweight geopolymer concrete could be produced using POC [26].

### 3.2. POC in Conventional Structural Elements

#### 3.2.1. POC as a Coarse Aggregate

The application of commercial aggregates is minimized due to high production costs emanating as a result of excessive raw materials and energy consumption. They also increase the dead weight of structures. Therefore, POC, as a porous and lightweight material that contains a high volume of solid waste materials, can be used to produce structural lightweight aggregate with potential for high-strength and good-workability concrete. POC density is said to be less than that of normal aggregate [50]. Even though substituting normal-weight coarse aggregate with POC wrecks the splitting tensile strength and modulus of elasticity, it improves the concrete’s compressive strength [51].

The physical properties of POC aggregate have a notable influence on the produced concrete properties. An equivalent of 1 m^3^ of soil is saved when 1 m^3^ of POC aggregate is utilized for concrete production instead of being discarded in a landfill. This would substantially lead to a safer and more productive climate [45]. CO_2_ emissions were said to have decreased by 20% when natural aggregate was totally replaced with POC coarse aggregate [52]. POC aggregates are lightweight and porous by nature, and they contribute to the reduction in concrete structural density [15]. POC increases a concrete mixture’s porosity and permeability. A compressive strength reduction of about 65% was recorded at full POC replacement. Nonetheless, concretes with lower strength could be used for the construction of pedestrian trails and walkways [53]. POC aggregate’s crushing value is three times less than that of gravel aggregate, thereby indicating higher energy consumption [35]. Having a density of 1990.33 kg/m^3^, POC aggregates are ideal for use in lightweight concrete mix proportions [54].

An experimental investigation was carried out on concrete substituted by POC as a filler and an aggregate material for high-strength concrete (HSC) creation. The permeable nature and uneven form of POC coarse aggregate had a negative impact on the fresh concrete mix’s workability. Nonetheless, adding POC powder as a filler improved the workability. Adding POC powder in POC concrete mixes improved the compressive, splitting tensile, and flexural strengths by 0–13%, 2–10%, and 1–9%, respectively compared to POC mix without POC powder. According to the rapid chloride permeability test (RCPT) carried out, both POC concrete mixes, with and without POC powder, had a strong resistance to chloride penetration with very low permeability at <100 °C [22].

**Table 4:** Physical properties of POC.

Size of Aggregate (mm)	Bulk Dry Density (kg/m^3^)	Saturated Density (kg/m^3^)	Specific Gravity	Water Absorption (%)	Aggregate Impact Value	Aggregate Crushing Value (%)	Fineness Modulus	Moisture Content (%)	Los Angeles Abrasion (%)	Compressive Strength (MPa) @ 28 Days	Splitting Tensile Strength (MPa)	Flexural Strength (MPa)	Ref.
‒	2050	2076	‒	‒	‒	‒	‒	‒	‒	57	‒	‒	[50]
5‒14	823	‒	1.62	4.43	‒	18.04	‒	‒	26.21	>30	‒	‒	[49]
‒	1471	65	‒	3.91	‒	‒	5.88	‒	‒	57.8	3.79	‒	[55]
4.75–10	732	‒	1.81	4.35	‒	56.44	‒	0.28	‒	55.2–83.5	‒	4–7.8	[22]
5‒14	781.08	1769.2	1.82	4.35	25.36	18.08	27.09	0.07	27.09	30.9	2.29	‒	[56]
‒	860	‒	1.69	7	36.3	21.2	‒	‒	23.9	‒	3.05–3.31	4.48–5.38	[57]
‒	1419	1875–1995	1.9	4.23	26.01	18.04	4.99	‒	27.08	50–60	3.2–4.6	‒	[58]
‒	782	‒	1.8	3.56	‒	‒	6.32	‒	‒	61.67	‒	‒	[16]
4.75–14	732	‒	1.73	3 ± 2	‒	56.44	‒	0.5	‒	33.01–39.32	2.61–3.28	3.75–4.42	[20]
4.75–9.5	732	‒	1.88	3 ± 2	‒	56.44	‒	‒	‒	3.43–9.52	‒	‒	[53]
5‒14	732	‒	1.73	3 ± 2	‒	56.44	‒	1 ± 0.5	‒	33–49	‒	‒	[27]
5–12.5	781.08	‒	1.82	4.35	25.36	‒	‒	‒	27.09	46	‒	‒	[59]
‒	817	‒	1.92	‒	‒	‒	2.6	1.3	38.7	‒	‒	‒	[54]
‒	568	‒	1.75	5.67	27.31	‒	‒	0.08	25.05	‒	‒	‒	[60]
‒	793	‒	1.76	4.67	48.6	47.9	‒	‒	‒	‒	‒	‒	[41]
‒	‒	‒	‒	‒	‒	‒	‒	‒	‒	27.51	‒	2.54	[18]
<4.75	811	‒	2.15	5.75	‒	‒	‒	0.11	‒	‒	‒	‒	[22]
<5	1118.86	‒	2.01	26.45	‒	‒	3.31	0.11	‒	‒	‒	‒	[56]
<5	811	‒	2.15	10 ± 5	‒	‒	‒	0.5 ± 0.25	‒	‒	‒	‒	[44]
‒	918	‒	1.98	‒	‒	‒	2.3	1.27	‒	‒	‒	‒	[54]
≤4.775	‒	‒	1.92	3.3 ± 1	‒	‒	3.52	1.5 ± 0.5	‒	53	‒	‒	[28]
‒	113	‒	2.08	3.6	‒	‒	3.12	‒	‒	‒	‒	‒	[61]
≤4.75	835.2	‒	1.92	3.3 ± 1	‒	‒	3.52	1.5 ± 0.5	‒	‒	‒	‒	[23]
‒	1085	‒	1.94	9.77	‒	‒	2.6	0.27	‒	‒	‒	‒	[37]

POC concrete beams have been known to provide sufficient notice of impending failure by exhibiting traditional structural ductile behavior. At service loads, the crack width (0.24–0.3 mm) of a POC concrete beam was found to be within the BS8110 overall permissible value for durability requirements [62]. In an oil palm shell (OPS) lightweight concrete, OPS aggregates were partly replaced with POC coarse aggregates from 0% to 50%. The slum value, density (2–4%), compressive strength, and modulus of elasticity (18–24%) of the OPS concrete increased as POC coarse aggregate was introduced into the mix. Moreover, at 20–50% POC coarse aggregate addition, grade 35 OPS concrete was upgraded to grade 40. As a result, it could be classified as a high-strength lightweight concrete [57]. In a related study, the authors reported a positive impact on workability, UPV, and compressive strength. The highest compressive strength of ~63 MPa (about 43% higher than the control mix) was obtained for the OPS:POC mixture. This may be due to efficient POC and mortar interlocking. With maximum obtainable stress between 0.00173 and 0.00401 higher than normal-weight concrete (NWC), the OPS:POC mixture could have better shrinkage and crack resistance capacity. Furthermore, a 2.5-fold rise in elasticity modulus could remarkably control deflection [62].

POC aggregate could be used to develop high-strength lightweight concrete with a 28 day compressive strength of 50–60 MPa and an oven-dry density of 1875–1995 kg/m^3^. In full water curing and air-drying curing conditions, equivalent compressive strengths were recorded, showing that POC lightweight concrete was not too sensitive to the curing method. The study suggested the use of regular sand with a nominal grain size of no more than 2 mm. This improved the elastic modulus of the concrete [57]. Ultrasonic pulse value (UPV) tests for POC concrete were good with a compressive strength and hardened density of 33–49 MPa and 2074–2358 kg/m^3^ at 28 days, respectively. At 10% POC replacement for coarse aggregate, grade 40 concrete was obtained. However, increasing the POC replacement ratio with coarse aggregate reduced the concrete workability. The advantage of applying POC as a lightweight aggregate is to decrease the dead load of concrete structures by up to 35% without much loss in structural strength. This decrease in dead load can save construction costs without compromising structural integrity. Therefore, applying lighter waste materials such as POC can greatly reduce concrete costs, due to its low cost of 0.020 MYR per kg. This will go a long way toward reducing the need for non-sustainable natural resources. For structural applications, the shear failure mode of POC concrete beams was found to be close to that of regular weight concrete beams, in line with ASTM:C330 [44].

Despite its higher porosity, self-compacting lightweight concrete (SCLWC) has strong UPV values. The tensile splitting strength, compressive strength, and flexural-to-compressive strength ratio also met the strength requirements for SCLWC [41]. Therefore, SCLWC is classified as a form of lightweight concrete with a high strength because of its 28 day compressive strength >40 N/mm^2^. As an actively mobilized material, POC was also able to amplify the filling and passing ability of self-compacting concrete. The concrete showed less segregation resistance due to low POC coarse density. Although obtained density values were in an acceptable range, coarse aggregate replacement with POC in SCLWC reduced the density in oven-dry and saturated surface-dry conditions by 16% and 18%, respectively.

Lightweight aggregate concretes made of POC with 12% less dead load compared to the conventional concrete mix showed an acceptable splitting tensile strength and workability without any segregation or floating at an average water-to-cement ratio. Interestingly, even after 28 days of curing, POC concretes did not achieve their maximum strength [55]. Testing the efficiency of POC in concrete slabs, the mechanical interlock (m) and friction (k) between the steel and concrete were found to be 117.67 N/mm^2^ and 0.0973 N/mm^2^, respectively. It was also discovered that the horizontal shear-bond strength and structural behavior were satisfactory, nearly comparable with conventional concrete slabs; thus, it could be used for composite slab construction. Compared to conventional concrete slabs, POC concrete slabs possess a reduction in weight of 18.3% [56]. Under absolute air, water, and 3 days water curing, the abrasion resistance and strength properties of concrete comprising POC coarse aggregate were investigated. The compressive strength of POC concrete cured in air and in water for 3 days displayed comparable behavior, with a maximum loss in strength of about 5% and an acceptable abrasion resistance. Interestingly, abrasion resistance was improved when cured in full water [52]. Air curing application in a tropical environment permits POC concrete to achieve the desired strength due to the surroundings high humidity. However, water curing is the most appropriate curing method for POC light aggregate concrete, because it contains enough water to ensure proper hydration and pozzolanic reactivity [60].

#### 3.2.2. POC as a Fine Aggregate

POC has, in recent times, been used as a partial replacement of fine aggregates in structural elements. This is possible due to the grading features and particle size distribution similarities between sand and POC fine aggregate [20,63]. The particle size distribution of POC ranging from 100–400 mm indicates its suitability for use as fine aggregates. A study by [18] found the compressive and flexural strength of concrete to increase upon replacing sand with POC. The study further confirmed that fine aggregate replacement with POC had no remarkable impact on compressive strength. However, it decreased concrete workability [44].

Sand was totally replaced by POC in a mortar designed using the volume-based approach. At 28 day curing, 41 MPa was recorded as the compressive strength of the mortar. POCS aids the gain of early-stage strength development (up to 77%). With 4.09 km/s POC mortar velocity, well-compacted specimens were obtained. The poriferous structure and rough nature of POC aided in the formation of a stronger bond with cement paste. The price of the mortar could be reduced by 16% when POC was utilized [23]. When POC was partially replaced with sand from 0–40% by weight of sand to investigate its effect on fly ash cement sand brick engineering properties, it was found that up to 30% POC usage enhanced the brick strength due to the pozzolanic effect of the fine clinker. Calcium hydroxide and silicon dioxide were responsible for the pore refinement and higher brick strength development [43]. Replacing OPC with fine aggregate increased the mortar sorptivity, as well as initial and final water absorption, because of its high porosity. OPC replacement changed the cement mortar’s thermophysical properties. At 100% sand replacement, compressive strength development (7 days to 28 days) was higher than samples containing lesser amounts of OPC. Under the same conditions, the specific heat capacity of mortar was boosted by ~41%. Thermal conductivity and diffusivity were lessened by 72% and 76%, respectively. This shows that OPC-replaced mortar has the potential to lower heat transfer and energy consumption in buildings [64].

In a related study at 100% sand replacement, it was reported that POC fine has the potential to produce 86% and 78% compressive strength at 28 and 56 days curing, respectively, providing almost 97% durability when compared to the conventional mix. POC fine durability showed a satisfactory outcome with good resistance to corrosion risk. POC fine is capable of lowering the carbon emissions of mortar by 50%. Moreover, POC fine can improve the engineering economic index and engineering environmental index by 11% and 95%, respectively. Life-cycle impact assessment (LCIA) showed POC’s potential to encourage a healthier and safer community with a substantial reduction in ecotoxicity [30].

Incorporating POC sand in OPS concrete is beneficial to reduce its sensitivity due to a lack of curing. OPC was used as a replacement for sand at 0–50% in an oil palm shell (OPS) lightweight aggregate concrete. It was discovered that the replacement does not affect the drying shrinkage strain. A high percentage of POC replacement increased the water absorption of the concrete. The concrete was proven to possess high splitting tensile strength [61]. In comparison with normal mining sand, POC fine aggregates have lower density and higher water absorption. Surprisingly, the slump value of concrete containing 25% POC fine showed good workability. The POC fine-replaced concrete was classified as having high strength because 69–76 MPa was obtained as the compressive strength for 28 day curing. Specifically, 12.5% POC fine replacement in concrete was said to be practical and cost-effective [65].

#### 3.2.3. POC Powder

**Figure 9:**
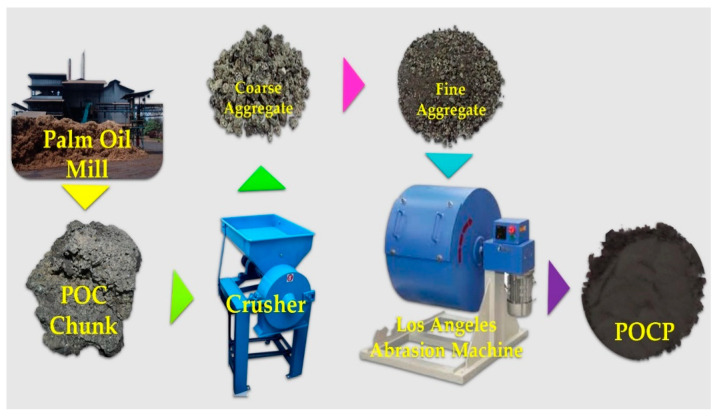
Preparation of POC powder [34].

The process of POCP preparation is depicted in Figure 9. It begins with obtaining POC chunk from a palm oil mill and subsequently putting it in an oven for 24 h at 105 °C to remove moisture. The POC powder is then obtained by crushing and grinding the dry POC to sizes below 2.36 mm for ~8 h in a controlled ball mill (Los Angeles abrasion machine) at 150 RPM with a 5:1 ball-to-powder mass ratio for utilization as a cementitious material. The crusher is used to obtain the coarse fine form of POC. The powder/fine material was then sieved using a BS sieve size 75 mm, and the resulting homogeneous filtrate material was termed “POCP”.

It has been confirmed by several authors through microstructure analysis that POCP particles are blackish in color, are irregular in shape, and contain small pores with fibrous materials present. SiO_2_, Al_2_O_3_, Fe_2_O_3_, MgO, and CaO are the major components found in POC powder with oxide composition >71.09%. This proves that the powder satisfies the chemical requirement of Class F fly ash. POCP and cement generally have similar fineness. However, the suitability of using them in concrete relies on their pozzolanic activity [22].

The strength activity index result proved that POCP is a pozzolanic material [66]. To ensure that the required workability can be attained when used for partial replacement of cement in mortars, POCP as a pozzolanic material would require more water. The crystallinity index of quartz in POC powder utilized by [21] was 0.97, indicating partial disorderliness of quartz and pozzolanic reactivity of the powder. The major component in POCP present in quartz and cristobalite phases at 2θ angles of 26.87° and 20.45°, respectively, is SiO_2_. A significant hump in XRD pattern from 10 °C to 35 °C demonstrates the presence of an amorphous fraction that is reactive due to pozzolanic activity [67].

The addition of POC powder to replace cement and quarry dust greatly increased the fresh and hardened density and compressive strength of produced blocks. Classified as thermally efficient and lightweight blocks, the properties of the produced blocks met the required thresholds and were higher than those of the common stabilized compressed earth blocks [37]. The use of POCP for cement replacement at about 40% in a cement–lime masonry mortar is recommended on the basis of the fresh density, consistency, and air content requirements. The split tensile strength at 90 days of curing was greatly improved due to the pozzolanic reactivity of POCP at longer duration. The flexural bond strength of the POCP mortar attained about 70% of that of the control mortar. It also reduced the carbon footprint by 32% and cost by 20%, as well as saved a reasonable amount of energy [34].

A study attempted to investigate the durability performance and microstructure behavior of masonry mortars where POCP was used for cement replacement. With a compressive strength of 12.5 MPa, 40% cement replacement appeared to be a reliable mortar in terms of durability front with similar 28 day drying shrinkage to the control mortar mix. The mixture possessed extremely good electrical resistivity [67].

POC powder significantly enhances concrete compactness. At a 15% increment, it improved the modulus of elasticity by up to 60% as compared to normal concrete. This could be attributed to concrete stiffness enhancement. At the same increment, the highest splitting tensile and flexural strengths in the range of normal weight concrete were recorded. Furthermore, 15% and 30% strength enhancements were obtained for flexural and compressive strengths (65 MPa). The study also found that, when utilizing POC powder at ~15–20% as a filler or cementitious material in producing grade 45 lightweight concrete, CO_2_ was reduced [25]. In a similar study trying to improve concrete strength, the authors used varying proportions of nano-palm oil clinker powder (NPOCP) for cement replacement. It was discovered that, as NPOCP content was increased in the concrete mix, density decreased. This is because cement has a higher specific gravity than NPOCP. However, an increase in NPOCP content increases concrete workability. The highest and lowest compressive values were obtained at 10% and 40% NPOCP replacement levels [37].

A POCP replacement level of up to 30% enhanced the resistance of recycled aggregate-based concrete to water absorption, and the risk of corrosion decreased to a “moderate” level after a 90 day curing period. In terms of compressive strength, the optimal replacement level of POCP to attain a satisfactory result was 20% in comparison with the normal mix [27].

In a study by [20], the surface voids of POC coarse were filled and coated with POCP as a filler material. This mixture could decrease the quantity of aggregates derived from primary sources that are continuously exploited. It also increases the paste content necessary to make the mixes more cohesive. A notable increment (5–25% higher) in flexural strength was attained as compared to the POC concrete, with a 20–30% increase attained for compressive strength. However, supplementing POCP led to a decrease in water absorption value by decreasing the pore size, thereby producing highly densified paste. Specimens that contain POCP were reported to exhibit greater chloride-ion resistance.

POC powder can reduce the cost of mortar by 41%, save 3.3% of cement production, and achieve 52% carbon emission reduction. Specifically, 50% POC powder-replaced mortar could achieve 70% strength and 60% structural efficiency as compared to normal mortar [35]. The pozzolanic reactivity, microstructure properties, and strength activity index result confirmed that POC powder has pozzolanic property and is good for utilization in cement-based applications.

### 3.3. POC in Wastewater Treatment

Domestic and industrial activities discharge wastewater containing high concentrations of various contaminants into water bodies [68,69]. Wastewater, usually full of contaminants, is considered as any water that is not safe for the intended use [70]. Wastewater as a hazardous substance/material is a byproduct resulting from human activities. However, it is a source of chemical and thermal energy [71]. Industrial operations in different mining fields, battery manufacturing, tannery, smelting, electroplating, textile, leather, petroleum processes, etc. are described as the major sources of wastewater [72,73]. Surface runoff, sewer infiltration, and poor management of urban solid waste also generate wastewater [74]. Discharging all these without treatment into watercourses exhausts the good quality of freshwater water bodies. Wastewater is known to contain toxic pollutants such as heavy metals and organic substances (dyes, PAHs, etc.) posing a great environmental threat to all living organisms [75,76]. Therefore, a reduction in effluent quantity and an improvement in quality would have major positive effects on land use and human health [77]. To achieve that, compliance with acceptable limits is required prior to discharging effluents into the environment. Researchers have engaged in developing safe, functional, cost-effective, and appropriate wastewater treatment technologies to improve the ecosystem, lessen pollutants’ detrimental effects, and minimize the risk of global warming and climate change. Unfortunately, some technologies and materials have shortcomings. As a result, it is imperative to develop safe, cost-effective, and long-lasting wastewater treatment materials [78].

POC as a waste material has recently been utilized by several researchers for wastewater treatment using techniques such as adsorption [79] and biological systems. Arsenic adsorption with palm oil clinker sand (POCS) was studied by [80]. They found that pH, arsenic concentration, POCS (mg), and temperature were the four significant variables controlling arsenic adsorption. Similar to several other adsorbents, the solution’s initial pH portrays the most prominent influence on adsorption. Water absorption, fitness modulus, and specific gravity were said to be the POC properties responsible for arsenic adsorption and process stability. Furthermore, the rich microporous structure and surface functional groups of POCs play a vital role in arsenic adsorption.

In a conventional activated sludge system, POC acted as a submerged attached growth medium for the treatment of domestic wastewater. Performance efficiency of the POC in the extended aeration system (EAS) was evaluated by COD, TSS, MLSS, and MLVSS. When comparing the performance of the POC submerged system to a biological activated sludge system, it could be concluded that using POC as an attached growth system can reduce the organic contaminant in effluent discharge [81].

POC as a filter medium in a sequence batch reactor system is capable of extending its useful life and reducing the demand for manufacturing new and sustainable media. In a comparative analysis between two SBR reactors with and without POC as a submerged fixed medium, the former had a higher ammonia removal efficiency of about 90% compared to the latter’s of 85% [82]. In a related study treating domestic wastewater in an SBR system, the average removal rates for ammonia and COD were 0.001 mg ammonia/mg MLVSS and 0.0069 mg COD/mg MLVSS, respectively. This amounted to 90% and 70% removal efficiencies for ammonia and COD, respectively [83].

### 3.4. Soil Stabilization

Deep foundations, specifically for soft soil, have been a problem for long time. This pushed geotechnical engineers to opt for lightweight concrete piles (LCPs) due to their peculiar properties such as low density, surface roughness, low strength, and high porosity. Different materials have been utilized to improve the concrete pile properties for performance enhancement. POC-incorporated concrete pile (p-LCP), foamed concrete pile (f-LCP), and normal concrete pile (NCP) for a floating foundation were investigated by conducting static and dynamic load tests. The findings revealed that higher compressive stress and driving resistance values were obtained for p-LCP and f-LCP when compared with NCP. According to a correlation of the compressive stress and driving resistance values of p-LCP and f-LCP with the pile ultimate load carrying capacity, the applied load for p-LCP and f-LCP could be increased by 4.5% and 27.3%, respectively. The driving resistance could also be increased by 27.6% and 16.5% for p-LCP and f-LCP, respectively. Therefore, the study concluded that p-LCP or f-LCP are better than NCP for deep foundations of particular structures in soft soil [84].

### 3.5. Highway Construction Material

In underdeveloped, developing, and developed nations, highway construction is vital for the wellbeing of citizens. This results in the overutilization of natural resources for construction. However, most of these resources have different environmental and financial implications for the immediate community. Therefore, a few researchers came up with the idea of using POC waste material for highway pavement construction application to help solve POC disposal issues.

**Figure 10:**
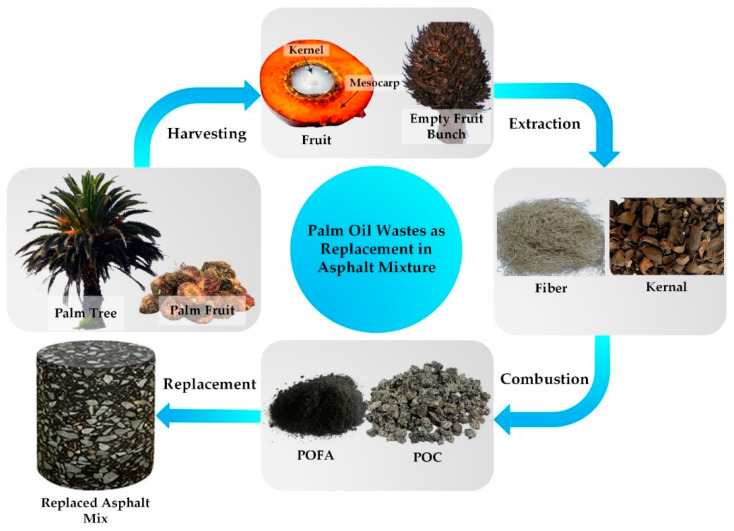
POC for asphalt mixture [19].

One study assessed the effect of using palm oil clinker (POC) as a substitute to fine aggregate for improving the mechanical properties of stone mastic asphalt (SMA) mixtures. The process of POC-incorporated SMA is depicted in Figure 10. It began with harvesting palm fruit from an oil palm tree and subsequently separating the fresh fruit from the empty fruit bunches. Fibers and kernels were carefully extracted from the empty fruit bunches to undergo a combustion process generating POFA and POC. A mixture of these two end products was utilized as the replacement material in an asphalt mixture.

The results proved the suitability of 100% POC replacement for fine aggregate in the SMA mixture, as it enhanced the drain down, resistance to moisture damage, resistance to rutting, and resilient modulus when compared to the control mixture. However, 40% and 60% replacements were considered optimal because of their outstanding mechanical properties. They also possessed higher indirect tensile strength in wet and dry conditions. Cantabro loss (durability performance) for POC-80 and POC-100 exceeded that of the control sample as all mixtures fulfilled the standard requirement of the maximum value (20%) for weight loss. The authors concluded that the use of POC as fine aggregates can greatly improve asphalt mix performance in flexible pavement construction [19].

In a related study by the same authors, using the Marshall mix design, POC had less of an effect on optimum binder content. The length of the elastic stage in the POC-replaced mixture was higher than that of the control mixture, thereby enhancing the elastic properties and making them more inclined toward plastic fracture. The fracture life of asphalt mixtures increased upon increasing the POC content in the mix. As a result, the asphalt mixtures were strong and stiff enough to withstand permanent deformation following traffic loads [85].

A study undertaken by [29] employed a high shear mixer to determine the appropriateness of utilizing palm oil clinker fine (POCF) as bitumen modifiers through material characterization tests. The impact of modification mixing parameters was also evaluated. The results from characterization confirmed its pozzolanic property, thus suggesting the feasibility of utilizing it as a bitumen modifier. The optimum mixing parameters obtained were 900 rpm at 160 °C for 30 min with 6.3% POCF as the optimum dosage. The study gathered that the incorporation of POCF enhances conventional bitumen properties [63].

### 3.6. POC as a Catalyst

In the downstream petrochemical industries, ethylene (C_2_H_4_) is one of the most highly sought raw materials. C_2_H_4_ is a primary precursor for surfactant fabrication, plastic manufacturing, and polyethylene production. Rather than landfilling POC, one study attempted the valorization of silica-rich POC into POC-derived SBA-15 (POC-SBA-15) catalysts and modulation of its surface acidity for C_2_H_4_ production via ethanol dehydration. With commercial SBA-15 as reference, the successful fabrication of POC-derived SBA-15 was validated. The specific surface area, pore volume, and pore size of commercial SBA catalysts were 642 m^2^/g, 0.83 cm^3^/g, and 8.02 nm, respectively, whereas those of POC-SBA-15 were found to be 537 m^2^/g, 0.71 cm^3^/g, and 7.84 nm, respectively. It could be observed that commercial SBA-15 had the highest surface textural properties. Although the POC-SBA-15 catalysts had a satisfactorily high catalytic area, they exhibited lower surface textural properties, as the mesoporous structure of POC-SBA-15 catalysts was partially covered by impurities of POC-Na_2_SiO_3_. Findings also revealed that the commercial SBA-15 had strong acidity, while POC-SBA-15 exhibited an enriched weak–moderate acidity. Despite the utilization of alternative sodium silicate (POC-Na_2_SiO_3_), TEM revealed that POC-SBA-15 catalysts preserved the structure of SBA. The optimal conditions of 400 °C temperature, 50 wt.% ethanol concentration, and 16 mL/g·h LHSV were determined for ethanol dehydration over POC-SBA-15(5) with the lowest strong and highest weak acidity. The POC mix catalyzed the process for up to 105 h. The practicality of POC as an alternative silica source for SBA-15 preparation was affirmed by the similar morphology between the commercial SBA-15 and POC-SBA-15 catalysts [42].

So far, there has been a single study concerning the valorization of POC-derived SBA-15. Therefore, to justify their findings, this review made a comparison with other similar silica-rich waste-derived SBA-15 catalysts found in the literature. SBA-15 was formed by a research team that worked on the synthesis of silica-based material from waste palm oil fuel ash (POFA). The BET surface area, pore volume, and pore diameter obtained were 330 m^2^/g, 0.33 cm^3^/g, and 4.52 nm, respectively. It was found that synthesized SBA-15(POFA) had good physicochemical properties and performed well in catalysis. The appearance of mesostructure characteristics, as attested by low-angle XRD, N_2_ adsorption–desorption isotherms, and transmission electron microscopy (TEM), demonstrated the successful synthesis of SBA-15 using a silica originator extracted from POFA. According to the current literature, used for comparative purposes, SBA-15 synthesized from tetraethyl ortho silicate (TEOS) had the largest surface area (699 m^2^/g) [86], trailed by sodium silicate SBA catalyst with 332 m^2^/g [87] and POFA-rich silicate with 330 m^2^/g. It was noted that the surface area of the SBA-15 synthesized using POFA sodium silicate was similar to that of commercial sodium silicate. This may be due to the chemical structure similarity of both silica sources. Similarly, the significant variation in TEOS silica origins could be attributed to the rising silica purity and TEOS’s different chemical structure. SBA-15(POFA) exhibited type IV isotherms with a clear H1 type hysteresis loop, corresponding to the SBA-15’s regular mesoporous characteristics [88].

The lignin-like fraction of biomass derived from green compost was immobilized on a silica support (SBA-15) and the physicochemical properties of the resulting hybrid material (BBS–SBA) were investigated. The catalyst had a surface area of 845 m^2^/g. The BBS–SBA hybrid is a well-characterized and stable material with unique characteristics for the removal of various species from aqueous solutions. Because of the presence of several dissociable groups at the BBS–SBA surface, the hydrophilic surface enabled effective interaction with charged substances in the presence of inorganic ions. Because of the synergy between Rhodamine B/Orange II adsorption and photocatalytic degradation, the manufacturing of hydroxyl radicals during BBS–SBA irradiation suggests a possible greater efficiency in organic contaminant removal [89].

### 3.7. POC as a Bio-Filler

To improve the mechanical strength, water resistance, and fire protection performance of steel structures, it is essential to use appropriate and cost-effective materials as bio-fillers in solvent-borne intumescent coatings. To that effect, waste byproducts such as chicken eggshells (CES), rice husk, and POC have been used to lessen the use of synthetic fillers. To produce intumescent coatings, POC and hybrid fillers are homogeneously mixed with an acrylic binder and subsequently blended with flame-retardant additives. POC has the advantages of large volume availability and direct usage without further processing.

Hybrid fillers with POC were blended with the proper amount of additives and acrylic binder in a related investigation by [90] to make intumescent coatings. When compared to specimens with hybrid fillers, specimens using POC as a solo filler considerably improved the fire protection performance of the intumescent coating, with a 10% temperature difference. Because of the limited solubility of Al(OH)_3_ in water, specimens using POC/Al(OH)_3_/TiO_2_ considerably increased the coating’s water resistance, but specimens with Mg(OH)_2_ exhibited higher mechanical strength due to the strong link that exists between the acrylic binder/Mg(OH)_2_ filler and metal surface.

## 4. Pretreatment of POC for Performance Enhancement

### 4.1. Effect of Hydrochloric (HCl) Acid and Magnesium Sulfate (MgSO_4_) Attack

The effect of hydrochloric acid and magnesium sulfate attack on POC-supplemented concrete was evaluated by [32]. The outcome proved that 30% POC addition minimizes concrete deterioration and loss in compressive strength when dipped in an HCl solution, exhibiting 30 MPa strength after 90 days curing. The concrete containing POC showed higher performance against deterioration, as well as mass and strength loss. This could be due to the low quantity of calcium hydroxide, well known as susceptible to acid attack. However, when the concrete was exposed to MgSO_4_ attack, fewer micro-cracks were seen.

### 4.2. Effect of Thermal and Chemical Treatment on POC Structural Elements

The fire resistance of any structural element greatly depends on the stability of concrete ingredients at elevated temperatures. Therefore, researchers usually conduct thermal activation to evaluate its effect on physical and mechanical properties, crystalline structure, minerals, organic carbon content, morphology, and chemical composition. Investigating these properties in necessary as they directly or indirectly affect the structural elements compressive strength. This is why Karim et al. [33] studied the effect of temperature on the microstructure change and compressive strength of cement paste incorporated with POC. It was reported that thermal activation at 600 °C and 800 °C for a duration of 3 h yield higher residual compressive strength for the POC specimen than that obtained for the OPC specimen. This could mainly be due to the pozzolanic reaction of the POC specimen when heated at elevated temperature. Furthermore, C-S-H gels were more stable in POC containing cement paste after exposure to an elevated temperature. This signified that the POC-incorporated specimen had higher fire resistance. Crack formation was also higher on the OPC paste surface, which is an indication of the superiority of POCP in making fire-resistant concrete.

In a related study by the same authors [91], 580 °C for 3 h was proven to be the appropriate condition for the thermal activation effect on POCP, as the compressive strength of mortar was significantly increased, the organic carbon content in POC was reduced, and the inorganic oxide content increased, with an increment rate of 3.4%, 3.5%, and 3.4% for SiO_2_, Al_2_O_3_, and Fe_2_O_3_, respectively. Porosity reduced as fibers were eliminated and POC color transformed from black to gray. It was also discovered that thermal activation has no significant influence on POC crystalline structure. Therefore, it was proven that thermal treatment can enhance POC pozzolanic reactivity by elevating the maturing process of hardened specimens and unburned carbon removal.

In order to investigate the pozzolanic reactivity of POC powder by chemical pretreatment, the powder was replaced at 2.5–15.0% by weight of cement for pretreated and untreated POC powder in mortar mixtures. POC impregnation with a low HCl acid concentration was able to enhance its pozzolanic reactivity through the hike of active silica proportion and reduced impurities and traces of metallic elements. The combination of 0.1 M HCl acid and 1 h of impregnation time was selected as the optimum pretreatment parameters. The strength activity index increased in the hardened mortar up to 7.5% cement content replacement with pretreated POC. The authors also concluded that the pretreatment process could enhance the pozzolanic reactivity of POC powder by up to 170%, increase the proportion of amorphous silica by up to 9.6%, and contribute more to the strength development of mortar compared to the untreated POC powder [38].

## 5. Conclusions

This review was designed to highlight the generation, disposal problems, properties, and composition of POC. The waste-to-resource potential of POC was greatly discussed starting with the application of POC in conventional and geopolymer structural elements such as beams, slabs, and columns made of concrete, mortar, or paste for coarse aggregates, sand, and cement replacement. Aspects such as the performance of POC in wastewater treatment processes, fine aggregate and cement replacement in asphaltic and bituminous mixtures during highway construction, a bio-filler in coatings for steel manufacturing processes, and a catalyst during energy generation were also discussed. The review also discussed the effectiveness of POC in soil stabilization and the effect of POC pretreatment for performance enhancement. During the study, it was discovered that POC utilization for intumescent coating can contribute to environmental conservation and reduce production cost. Furthermore, 37% of waste materials from the palm industry are used in the development of green concrete; with the significant rise in global vegetable oil production, this is projected to grow even further. Thus, the incorporation of POC as an alternative raw material for concrete work, with or without pretreatment, will help to maintain the construction industry’s long-term viability. POC has been shown to function in a variety of concretes, including self-compacting, natural, lightweight, pervious, and supplementary cementitious materials. The present review could be used for researchers’ foundational awareness to motivate them to explore the high potential of utilizing POC for greater environmental benefits associated with lower cost when compared with conventional materials. Lastly, this review recommends future researchers to investigate the feasibility of utilizing micro, ultrafine, and nano POC powder for various applications that will promote environmental sustainability.

## Figures and Tables

**Table 2:** Chemical composition of POC.

State of POC	Chemical Composition (%)												Ref.
	CaO	SiO_2_	SO_3_	Fe_2_O_3_	Al_2_O_3_	MgO	P_2_O_5_	K_2_O	TiO_2_	Mn_2_O_3_	Na_2_O	Others	
Coarse	8.16	59.63	0.73	4.62	3.70	5.01	5.37	11.66	0.22	-	0.32	0.58	[16]
Fine	17.00	53.7	0.92	3.87	1.46	2.37	5.29	13.9	-	-	-	-	[29]
Fine	6.37	59.90	0.39	6.93	3.89	3.30	3.47	15.10	0.29	-	-	0.36	[30]
Fine	5.74	65.40	0.64	2.71	1.95	6.40	6.56	9.52	0.11	0.17	0.32	-	[19]
Fine	3.28	60.29	0.31	4.71	5.83	4.20	3.78	7.24	0.10	0.12	0.20	-	[23]
Powder	6.37	59.90	0.39	6.93	3.89	3.30	3.47	15.10	0.29	-	-	0.36	[20]
Powder	3.20	61.29	0.10	4.31	5.89	3.16	3.12	10.79	0.12	-	-	-	[31]
Powder	3.26	60.29	0.11	4.71	5.83	3.76	3.10	7.79	0.13	-	-	-	[22]
Powder	3.28	60.29	0.31	4.71	5.83	4.20	-	-	-	-	-	-	[32]
Powder	6.93	63.90	0.21	3.30	3.89	3.37	2.12	10.20	-	-	-	-	[21]
Powder	3.89	65.30	0.09	5.65	4.23	3.72	0.78	13.65	0.13	-	-	-	[33]
Powder	8.00	60.00	-	4.00	4.00	5.00	5.00	12.00	-	-	-	2.00	[25]
Powder	3.28	60.29	0.31	4.71	5.83	4.20	3.78	7.24	0.10	0.12	0.20	-	[34]
Powder	6.37	59.90	2.60	6.93	5.37	3.13	0.07	15.10	0.12	0.12	0.24	-	[35]
Powder	4.82	63.10	0.15	9.00	3.20	3.50	3.09	12.50	0.21	0.12	0.16	0.13	[36]
Powder	6.09	42.30		3.288	3.09	2.41	2.95	15.20	0.17	-	-	-	[37]
Powder	7.05	55.39	0.19	10.81	2.18	2.00	3.97	17.7		-	-	0.28	[38]

**Table 3:** SLR objectives and questions.

	Objectives	Questions
1	To identify the most common engineering and environmental application of POC and their properties	What are the distinguishing POC properties and the most common area of application in engineering?
2	To verify the performance of POC in different forms when applied for engineering designs and manufacturing	How do POC forms and their properties affect the efficiency of engineering designs and manufacturing?

## Data Availability

The data presented in this study are available from the corresponding author upon reasonable request.

## References

[1] Duque-Acevedo M., Belmonte-Urena L.J., Cortés-García F.J., Camacho-Ferre F. (2020). Agricultural waste: Review of the evolution, approaches and perspectives on alternative uses. Glob. Ecol. Conserv..

[2] Harun N.Y., Han T.J., Vijayakumar T., Saeed A., Afzal M. (2019). Ash deposition characteristics of industrial biomass waste and agricultural residues. Mater. Today Proc..

[3] Ukanwa K., Patchigolla K., Sakrabani R., Anthony E., Mandavgane S. (2019). A review of chemicals to produce activated carbon from agricultural waste biomass. Sustainability.

[4] Lee Z.S., Chin S.Y., Lim J.W., Witoon T., Cheng C.K. (2019). Treatment technologies of palm oil mill effluent (POME) and olive mill wastewater (OMW): A brief review. Environ. Technol. Innov..

[5] Meschini R., Eliseo D.D., Filippi S., Bertini L., Bizzarri B.M., Botta L., Saladino R., Velotti F. (2018). Tyrosinase-treated hydroxytyrosol-enriched olive vegetation waste with increased antioxidant activity promotes autophagy and inhibits the inflammatory response in human THP-1 monocytes. J. Agric. Food Chem..

[6] Cádiz-Gurrea M., Pinto D., Delerue-Matos C., Rodrigues F. (2021). Olive fruit and leaf wastes as bioactive ingredients for cosmetics—A preliminary study. Antioxidants.

[7] De Matteis V., Rizzello L., Ingrosso C., Rinaldi R., Research P. (2021). Purification of olive mill wastewater through noble metal nanoparticle synthesis: Waste safe disposal and nanomaterial impact on healthy hepatic cell mitochondria. Environ. Sci. Pollut. Res..

[8] Cheng Y.W., Chong C.C., Lam M.K., Ayoub M., Cheng C.K., Lim J.W., Yusup S., Tang Y., Bai J. (2020). Holistic process evaluation of non-conventional palm oil mill effluent (POME) treatment technologies: A conceptual and comparative review. J. Hazard. Mater..

[9] Jagaba A., Kutty S., Hayder G., Baloo L., Ghaleb A., Lawal I., Abubakar S., Al-dhawi B., Almahbashi N., Umaru I. (2021). Degradation of Cd, Cu, Fe, Mn, Pb and Zn by Moringa-oleifera, zeolite, ferric-chloride, chitosan and alum in an industrial effluent. Ain Shams Eng. J..

[10] Solarte-Toro J.C., González-Aguirre J.A., Giraldo J.A.P., Alzate C.A.C., Reviews S.E. (2021). Thermochemical processing of woody biomass: A review focused on energy-driven applications and catalytic upgrading. Renew. Sustain. Energy Rev..

[11] Nur M.M.A., Buma A.G.J. (2019). Opportunities and challenges of microalgal cultivation on wastewater, with special focus on palm oil mill effluent and the production of high value compounds. Waste Biomass Valoriz..

[12] Hii K.-L., Yeap S.-P., Mashitah M.D. (2012). Cellulase production from palm oil mill effluent in Malaysia: Economical and technical perspectives. Eng. Life Sci..

[13] Poh P.E., Wu T.Y., Lam W.H., Poon W.C., Lim C.S. (2020). Waste Management in the Palm Oil Industry: Plantation and Milling Processes.

[14] Saad M.S., Wirzal M.D.H., Putra Z.A. (2021). Review on current approach for treatment of palm oil mill effluent: Integrated system. J. Environ. Manag..

[15] Kanadasan J., Razak H.A. (2014). Mix design for self-compacting palm oil clinker concrete based on particle packing. Mater. Des..

[16] Jumaat M.Z., Alengaram U.J., Ahmmad R., Bahri S., Islam A.B.M.S. (2015). Characteristics of palm oil clinker as replacement for oil palm shell in lightweight concrete subjected to elevated temperature. Constr. Build. Mater..

[17] Mustapa S., Sulong N.R. (2017). Performance of palm oil clinker as a bio-filler with hybrid fillers in intumescent fire protective coatings for steel. Sains Malays..

[18] Ibrahim M.H.W., Mangi S.A., Ridzuan M.B., Burhanudin M., Jamaluddin N., Li K.H., Shahidan S., Khalid F.S., Arshad M.F., Jaya R.P. (2018). Compressive and flexural strength of concrete containing palm oil biomass clinker with hooked-end steel fibers. Int. J. Integr. Eng..

[19] Babalghaith A.M., Koting S., Sulong N.H.R., Karim M.R., AlMashjary B.M. (2020). Performance evaluation of stone mastic asphalt (SMA) mixtures with palm oil clinker (POC) as fine aggregate replacement. Constr. Build. Mater..

[20] Abutaha F., Razak H.A., Ibrahim H.A. (2017). Effect of coating palm oil clinker aggregate on the engineering properties of normal grade concrete. Coatings.

[21] Karim M.R., Hashim H., Razak H.A., Yusoff S. (2017). Characterization of palm oil clinker powder for utilization in cement-based applications. Constr. Build. Mater..

[22] Abutaha F., Razak H.A., Ibrahim H.A., Ghayeb H.H. (2018). Adopting particle-packing method to develop high strength palm oil clinker concrete. Resour. Conserv. Recycl..

[23] Darvish P., Alengaram U.J., Poh Y.S., Ibrahim S., Yusoff S. (2020). Volume based design approach for sustainable palm oil clinker as whole replacement for conventional sand in mortar. J. Build. Eng..

[24] Karim M.R., Yusoff S., Razak H.A., Chowdhury F.I., Zabed H. (2018). Heavy metals leaching behaviour assessment of palm oil clinker. Sains Malays..

[25] Ahmmad R., Alengaram U.J., Jumaat M.Z., Sulong N.R., Yusuf M.O., Rehman M.A. (2017). Feasibility study on the use of high volume palm oil clinker waste in environmental friendly lightweight concrete. Constr. Build. Mater..

[26] Kabir S.A., Alengaram U.J., Jumaat M.Z., Yusoff S., Sharmin A., Bashar I.I. (2017). Performance evaluation and some durability characteristics of environmental friendly palm oil clinker based geopolymer concrete. J. Clean. Prod..

[27] Alnahhal M.F., Alengaram U.J., Jumaat M.Z., Abutaha F., Alqedra M.A., Nayaka R.R. (2018). Assessment on engineering properties and CO2 emissions of recycled aggregate concrete incorporating waste products as supplements to Portland cement. J. Clean. Prod..

[28] Darvish P., Alengaram U.J., Poh Y.S., Ibrahim S., Yusoff S. (2020). Performance evaluation of palm oil clinker sand as replacement for conventional sand in geopolymer mortar. Constr. Build. Mater..

[29] Yaro N., Napiah M., Sutanto M., Usman A., Saeed S., Kaura J. (2021). Influence of modification mixing parameters on conventional properties of palm oil clinker fine (POCF)-modified bitumen. Mater. Today Proc..

[30] Kanadasan J., Razak H.A., Subramaniam V. (2018). Properties of high flowable mortar containing high volume palm oil clinker (POC) fine for eco-friendly construction. J. Clean. Prod..

[31] Karim M.R., Khandaker M.U., Asaduzzaman K., Razak H.A., Yusoff S. (2019). Radiological risks assessment of building materials ingredients: Palm oil clinker and fuel ash. Indoor Built Environ..

[32] Alnahhal M.F., Alengaram U.J., Jumaat M.Z., Alsubari B., Alqedra M.A., Mo K.H. (2018). Effect of aggressive chemicals on durability and microstructure properties of concrete containing crushed new concrete aggregate and non-traditional supplementary cementitious materials. Constr. Build. Mater..

[33] Karim M.R., Chowdhury F.I., Zabed H., Saidur M. (2018). Effect of elevated temperatures on compressive strength and microstructure of cement paste containing palm oil clinker powder. Constr. Build. Mater..

[34] Nayaka R.R., Alengaram U.J., Jumaat M.Z., Yusoff S.B., Alnahhal M.F. (2018). High volume cement replacement by environmental friendly industrial by-product palm oil clinker powder in cement–lime masonry mortar. J. Clean. Prod..

[35] Kanadasan J., Abdul Razak H. (2015). Utilization of palm oil clinker as cement replacement material. Materials.

[36] Hamada H., Alattar A., Yahaya F., Muthusamy K., Tayeh B.A. (2021). Mechanical properties of semi-lightweight concrete containing nano-palm oil clinker powder. Phys. Chem. Earth Parts A/B/C.

[37] Shakir A.A., Ibrahim M.W., Othman N., Mohammed A.A., Burhanudin M. (2020). Production of eco-friendly hybrid blocks. Constr. Build. Mater..

[38] Ismail A.H., Kusbiantoro A., Chin S.C., Muthusamy K., Islam M., Tee K.F. (2020). Pozzolanic reactivity and strength activity index of mortar containing palm oil clinker pretreated with hydrochloric acid. J. Clean. Prod..

[39] Jagaba A., Kutty S., Hayder G., Latiff A., Aziz N., Umaru I., Ghaleb A., Abubakar S., Lawal I., Nasara M. (2020). Sustainable use of natural and chemical coagulants for contaminants removal from palm oil mill effluent: A comparative analysis. Ain Shams Eng. J..

[40] Baloo L., Isa M.H., Sapari N.B., Jagaba A.H., Wei L.J., Yavari S., Razali R., Vasu R. (2021). Adsorptive removal of methylene blue and acid orange 10 dyes from aqueous solutions using oil palm wastes-derived activated carbons. Alex. Eng. J..

[41] Feen O.S., Mohamed R.N., Mohamed A., Khalid N.H.A. (2017). Effects of coarse palm oil clinker on properties of self-compacting lightweight concrete. J. Teknol..

[42] Cheng Y.W., Chong C.C., Cheng C.K., Ng K.H., Witoon T., Juan J.C. (2020). Ethylene production from ethanol dehydration over mesoporous SBA-15 catalyst derived from palm oil clinker waste. J. Clean. Prod..

[43] Muthusamy K., Budiea A.M.A., Mohsin S.M.S., Zam N.S.M., Nadzri N.E.A. (2020). Properties of fly ash cement brick containing palm oil clinker as fine aggregate replacement. Mater. Today Proc..

[44] Abutaha F., Razak H.A., Kanadasan J. (2016). Effect of palm oil clinker (POC) aggregates on fresh and hardened properties of concrete. Constr. Build. Mater..

[45] Kanadasan J., Razak H.A. (2015). Engineering and sustainability performance of self-compacting palm oil mill incinerated waste concrete. J. Clean. Prod..

[46] Jagaba A.H., Kutty S.R.M., Noor A., Birniwa A.H., Affam A.C., Lawal I.M., Kankia M.U., Kilaco A.U. (2021). A systematic literature review of biocarriers: Central elements for biofilm formation, organic and nutrients removal in sequencing batch biofilm reactor. J. Water Process. Eng..

[47] Yashni G., Al-Gheethi A., Radin Mohamed R.M.S., Arifin S.N.H., Mohd Salleh S.N.A. (2020). Technology. Conventional and advanced treatment technologies for palm oil mill effluents: A systematic literature review. J. Dispers. Sci. Technol..

[48] Nazari A., Riahi S., Bagheri A. (2012). Designing water resistant lightweight geopolymers produced from waste materials. Mater. Des..

[49] Malkawi A.B., Habib M., Alzubi Y., Aladwan J. (2020). Engineering properties of lightweight geopolymer concrete using palm oil clinker aggregate. Int. J..

[50] Aslam M., Shafigh P., Jumaat M.Z. (2016). Effect of replacement of oil-palm-boiler clinker with oil palm shell on the properties of concrete. AIP Conf. Proc..

[51] Chai L.J., Shafigh P., Mahmud H., Aslam M. (2017). Effect of substitution of normal weight coarse aggregate with oil-palm-boiler clinker on properties of concrete. Sains Malays..

[52] Ibrahim H.A., Razak H.A., Abutaha F. (2017). Strength and abrasion resistance of palm oil clinker pervious concrete under different curing method. Constr. Build. Mater..

[53] Ibrahim H.A., Razak H.A. (2016). Effect of palm oil clinker incorporation on properties of pervious concrete. Constr. Build. Mater..

[54] Nazreen M.S., Mohamed R.N., Ab Kadir M.A., Azillah N., Shukri N.A., Mansor S., Zamri F. (2018). Characterization of lightweight concrete made of palm oil clinker aggregates. MATEC Web Conf..

[55] Shafigh P., Chai L.J., Mahmud H.B., Nomeli M.A. (2018). A comparison study of the fresh and hardened properties of normal weight and lightweight aggregate concretes. J. Build. Eng..

[56] Mohammed B.S., Al-Ganad M.A., Abdullahi M. (2011). Analytical and experimental studies on composite slabs utilising palm oil clinker concrete. Constr. Build. Mater..

[57] Aslam M., Shafigh P., Jumaat M.Z., Lachemi M. (2016). Benefits of using blended waste coarse lightweight aggregates in structural lightweight aggregate concrete. J. Clean. Prod..

[58] Chai L.J., Shafigh P., Bin Mahmud H. (2019). Production of high-strength lightweight concrete using waste lightweight oil-palm-boiler-clinker and limestone powder. Eur. J. Environ. Civ. Eng..

[59] Huda M.N., Jumat M.Z.B., Islam A.S. (2016). Flexural performance of reinforced oil palm shell & palm oil clinker concrete (PSCC) beam. Constr. Build. Mater..

[60] Muthusamy K., Mirza J., Zamri N.A., Hussin M.W., Majeed A.P.A., Kusbiantoro A., Budiea A.M.A. (2019). Properties of high strength palm oil clinker lightweight concrete containing palm oil fuel ash in tropical climate. Constr. Build. Mater..

[61] Shafigh P., Mahmud H.B., Jumaat M.Z.B., Ahmmad R., Bahri S. (2014). Structural lightweight aggregate concrete using two types of waste from the palm oil industry as aggregate. J. Clean. Prod..

[62] Ahmmad R., Jumaat M.Z., Alengaram U.J., Bahri S., Rehman M.A., bin Hashim H. (2016). Performance evaluation of palm oil clinker as coarse aggregate in high strength lightweight concrete. J. Clean. Prod..

[63] Yaro N., Napiah M., Sutanto M.H., Usman A., Rafindadi A.D., Saeed S.M., Abdulrahman M. (2021). Evaluation of the impact of short-term aging on volumetric and Marshall properties of palm oil clinker fine modified asphalt concrete (POCF-MAC). J. Phys. Conf. Ser..

[64] Asadi I., Shafigh P., Hashemi M., Akhiani A.R., Maghfouri M., Sajadi B., Mahyuddin N., Esfandiari M., Talebi H.R., Metselaar H.S.C. (2020). Thermophysical properties of sustainable cement mortar containing oil palm boiler clinker (OPBC) as a fine aggregate. Constr. Build. Mater..

[65] Soofinajafi M., Shafigh P., Akashah F.W., Mahmud H.B. (2016). Mechanical properties of high strength concrete containing coal bottom ash and oil-palm boiler clinker as fine aggregates. MATEC Web Conf..

[66] Karim M.R., Hashim H., Razak H.A. (2016). Assessment of pozzolanic activity of palm oil clinker powder. Constr. Build. Mater..

[67] Nayaka R.R., Alengaram U.J., Jumaat M.Z., Yusoff S.B. (2018). Microstructural investigation and durability performance of high volume industrial by-products-based masonry mortars. Constr. Build. Mater..

[68] Ng J., Wong D., Kutty S., Jagaba A.H. (2021). Organic and nutrient removal for domestic wastewater treatment using bench-scale sequencing batch reactor. AIP Conf. Proc..

[69] Noor A., Kutty S., Baloo L., Almahbashi N., Kumar V., Ghaleb A. (2021). Bio-kinetics of organic removal in EAAS reactor for co-treatment of refinery wastewater with municipal wastewater. IOP Conf. Ser. Mater. Sci. Eng..

[70] Jagaba A.H., Abubakar S., Nasara M.A., Jagaba S.M., Chamah H.M., Lawal I.M., Chemistry T. (2019). Defluoridation of drinking water by activated carbon prepared from tridax procumbens plant (A Case Study of Gashaka Village, Hong LGA, Adamawa State, Nigeria). Int. J. Comput. Theor. Chem..

[71] Saeed A.A.H., Harun N.Y., Sufian S., Bilad M.R., Nufida B.A., Ismail N.M., Zakaria Z.Y., Jagaba A.H., Ghaleb A.A.S., Al-Dhawi B.N.S. (2021). Modeling and optimization of biochar based adsorbent derived from Kenaf using response surface methodology on adsorption of Cd2+. Water.

[72] Almahbashi N., Kutty S., Ayoub M., Noor A., Salihi I., Al-Nini A., Jagaba A., Aldhawi B., Ghaleb A. (2021). Optimization of preparation conditions of sewage sludge based activated carbon. Ain Shams Eng. J..

[73] Mustafa H.M., Hayder G., Jagaba A.H. (2021). Microalgae: A Renewable Source for Wastewater Treatment and Feedstock Supply for Biofuel Generation. Biointerface Res. Appl. Chem.

[74] Birniwa A.H., Abubakar A.S., Huq A.O., Mahmud H.N.M.E. (2021). Polypyrrole-polyethyleneimine (PPy-PEI) nanocomposite: An effective adsorbent for nickel ion adsorption from aqueous solution. J. Macromol. Sci. Part A.

[75] Saeed A.A.H., Harun N.Y., Nasef M.M., Al-Fakih A., Ghaleb A.A.S., Afolabi H.K. (2021). Removal of cadmium from aqueous solution by optimized rice husk biochar using response surface methodology. Ain Shams Eng. J..

[76] Hezam Saeed A.A., Harun N.Y., Sufian S., Bin Aznan M.F. (2020). Technology. Effect of Adsorption Parameter on the Removal of Nickel (II) by Low-Cost Adsorbent Extracted From Corn Cob. Int. J. Adv. Res. Eng. Technol..

[77] Jagaba A., Kutty S., Lawal I., Abubakar S., Hassan I., Zubairu I., Umaru I., Abdurrasheed A., Adam A., Ghaleb A. (2021). Sequencing batch reactor technology for landfill leachate treatment: A state-of-the-art review. J. Environ. Manag..

[78] Saeed A.A.H., Harun N.Y., Sufian S., Siyal A.A., Zulfiqar M., Bilad M.R., Vagananthan A., Al-Fakih A., Ghaleb A.A.S., Almahbashi N. (2020). Eucheuma cottonii Seaweed-Based Biochar for Adsorption of Methylene Blue Dye. Sustainability.

[79] Saeed A.A.H., Harun N.Y., Sufian S., Afolabi H.K., Al-Qadami E.H.H., Roslan F.A.S., Rahim S.A., Ghaleb A., Technology E. (2021). Production and characterization of rice husk biochar and Kenaf biochar for value-added biochar replacement for potential materials adsorption. Ecol. Eng. Environ. Technol..

[80] Rehman M.A., Yusoff I., Ahmmad R., Alias Y. (2015). Arsenic adsorption using palm oil waste clinker sand biotechnology: An experimental and optimization approach. Water Air Soil Pollut..

[81] Al-dhawi B.N., Kutty S.R., Almahbashi N.M., Noor A., Jagaba A.H. (2020). Organics removal from domestic wastewater utilizing palm oil clinker (POC) media in a submerged attached growth systems. Int. J. Civ. Eng. Technol..

[82] Chew Y., Yap Z., Kutty S., Ghaleb A., Almahbashi N. (2020). Removal of ammonia by palm oil clinker (POC) as submerged fixed media in sequence batch reactor (SBR) mode. IOP Conf. Ser. Mater. Sci. Eng..

[83] Kuan Y.Z., Kutty S.R., Ghaleb A.A. (2019). Kinetics Coefficient of Palm Oil Clinker Media for an Attached Growth Media in Sequencing Batch Reactor Mode. J. Ecol. Eng..

[84] Sulaeman A., Fulazzaky M.A., Haroen M., Bakar I. (2018). Field test results of palm oil clinker concrete pile and foamed concrete pile for floating foundation in soft soil. KSCE J. Civ. Eng..

[85] Babalghaith A.M., Koting S., Sulong N.H.R., Karim M.R., Mohammed S.A., Ibrahim M.R. (2020). Effect of Palm Oil Clinker (POC) Aggregate on the Mechanical Properties of Stone Mastic Asphalt (SMA) Mixtures. Sustainability.

[86] Charan P., Rao G.R. (2015). Textural and morphological studies of transition metal doped SBA-15 by co-condensation method. J. Chem. Sci..

[87] Rahmat N., Hamzah F., Sahiron N., Mazlan M., Zahari M.M. (2016). Sodium silicate as source of silica for synthesis of mesoporous SBA-15. IOP Conf. Ser. Mater. Sci. Eng..

[88] Abdullah N., Ainirazali N., Chong C., Razak H., Setiabudi H., Chin S., Jalil A.A. (2020). Effect of Ni loading on SBA-15 synthesized from palm oil fuel ash waste for hydrogen production via CH4 dry reforming. Int. J. Hydrog. Energy.

[89] Tummino M.L., Testa M.L., Malandrino M., Gamberini R., Bianco Prevot A., Magnacca G., Laurenti E. (2019). Green waste-derived substances immobilized on SBA-15 silica: Surface properties, adsorbing and photosensitizing activities towards organic and inorganic substrates. Nanomaterials.

[90] Mustapa S., Sulong N.R. (2017). Performance of solvent-borne intumescent fire protective coating with Palm oil clinker as novel bio-filler on steel. IOP Conf. Ser. Mater. Sci. Eng..

[91] Karim M.R., Hashim H., Razak H.A. (2016). Thermal activation effect on palm oil clinker properties and their influence on strength development in cement mortar. Constr. Build. Mater..

